# Enhanced Cartilage and Subchondral Bone Repair Using Carbon Nanotube-Doped Peptide Hydrogel–Polycaprolactone Composite Scaffolds

**DOI:** 10.3390/pharmaceutics15082145

**Published:** 2023-08-15

**Authors:** Jiayi Lv, Yilun Wu, Zhicheng Cao, Xu Liu, Yuzhi Sun, Po Zhang, Xin Zhang, Kexin Tang, Min Cheng, Qingqiang Yao, Yishen Zhu

**Affiliations:** 1 College of Biotechnology and Pharmaceutical Engineering, Nanjing Tech University, Nanjing 211816, China; 2 Department of Orthopaedic Surgery, Institute of Digital Medicine, Nanjing First Hospital, Nanjing Medical University, Nanjing 210006, China; 3 College of Pharmaceutical Sciences, Nanjing Tech University, Nanjing 211816, China

**Keywords:** carbon nanotube-doped, self-assembled peptide hydrogel, polycaprolactone, composite scaffolds, cartilage and subchondral bone repair

## Abstract

A carbon nanotube-doped octapeptide self-assembled hydrogel (FEK/C) and a hydrogel-based polycaprolactone PCL composite scaffold (FEK/C_3_-S) were developed for cartilage and subchondral bone repair. The composite scaffold demonstrated modulated microstructure, mechanical properties, and conductivity by adjusting CNT concentration. In vitro evaluations showed enhanced cell proliferation, adhesion, and migration of articular cartilage cells, osteoblasts, and bone marrow mesenchymal stem cells. The composite scaffold exhibited good biocompatibility, low haemolysis rate, and high protein absorption capacity. It also promoted osteogenesis and chondrogenesis, with increased mineralization, alkaline phosphatase (ALP) activity, and glycosaminoglycan (GAG) secretion. The composite scaffold facilitated accelerated cartilage and subchondral bone regeneration in a rabbit knee joint defect model. Histological analysis revealed improved cartilage tissue formation and increased subchondral bone density. Notably, the FEK/C_3_-S composite scaffold exhibited the most significant cartilage and subchondral bone formation. The FEK/C_3_-S composite scaffold holds great promise for cartilage and subchondral bone repair. It offers enhanced mechanical support, conductivity, and bioactivity, leading to improved tissue regeneration. These findings contribute to the advancement of regenerative strategies for challenging musculoskeletal tissue defects.

## 1. Introduction

Articular cartilage defects are frequently accompanied by subchondral flaws and can be easily caused by trauma, ageing, and disease, making cartilage healing a challenging task [1]. The lack of blood arteries and nerve tissue further complicates the repair process [2] In clinical practice, the treatment of cartilage defects remains a perennial challenge. Current clinical strategies involve microfracture, autologous chondrocyte transplantation, and autologous cartilage transplantation [3,4,5,6]. However, these treatments have limitations, such as the formation of fibrocartilage, poor effects of tissue integration, or a lack of available autografts [7,8]. Consequently, there is an urgent need for novel treatment strategies.

Tissue engineering has emerged as a promising approach for addressing cartilage defects [9,10]. The key step in this process is the development of a bone tissue engineering scaffold with osteogenic capacity. Specifically, scaffolds for cartilage defects should possess superior mechanical qualities to support the subchondral bone. Polycaprolactone (PCL), an FDA-approved material, has been widely used in cartilage regeneration due to its outstanding mechanical properties [11,12,13]. However, the hydrophobic nature of PCL scaffolds limits their biocompatibility, resulting in poor cell attachment and proliferation [14,15,16]. Current research focuses on improving PCL biocompatibility by incorporating bioceramics, polymers, and hydrogels [17,18,19,20].

Peptide hydrogels, known for their good biocompatibility, provide an extracellular microenvironment (ECM)-like environment that promotes cell attachment and proliferation [21,22]. In previous studies, a non-immunogenetic octapeptide (FEFEFKFK) was designed and synthesized [23,24]. This octapeptide can form self-assembling peptide hydrogel (SAPH) with a typical structure consisting of nano-sized β sheet stacks, also called FEK hydrogels for short. The self-assembling process is triggered by ionic interactions, van der Waals forces, hydrogen bond formation, and hydrophobicity [25]. The resulting peptide hydrogel exhibits excellent biocompatibility and has been used to support the attachment and proliferation of nucleus pulposus cells and chondrocytes [26,27]. Encapsulated chondrocytes in the peptide hydrogel demonstrated active proliferation and deposition of type II collagen, maintaining a round shape for an extended period [26]. 

Recent studies revealed that carbon nanotubes (CNTs) can facilitate both cartilage and subchondral bone regeneration [28,29,30]. CNTs are hollow tubes formed by twisted graphite sheets, providing a high specific surface area, nano-sized networks, and appropriate porosity for cellular material exchanges [31,32,33,34,35]. Additionally, CNTs possess good conductivity, which benefits the regeneration of cartilage tissues. Incorporating CNTs into PCL scaffolds can enhance cell inoculation efficiency, promote cell interaction, support adhesion and proliferation of bone marrow-derived mesenchymal stem cells (BMSCs), and facilitate osteogenic differentiation in vitro. However, high concentrations of CNT lead to their agglomeration, necessitating their integration with other commonly used materials to ensure safety and maximize their superior properties [36].

**Scheme 1:**
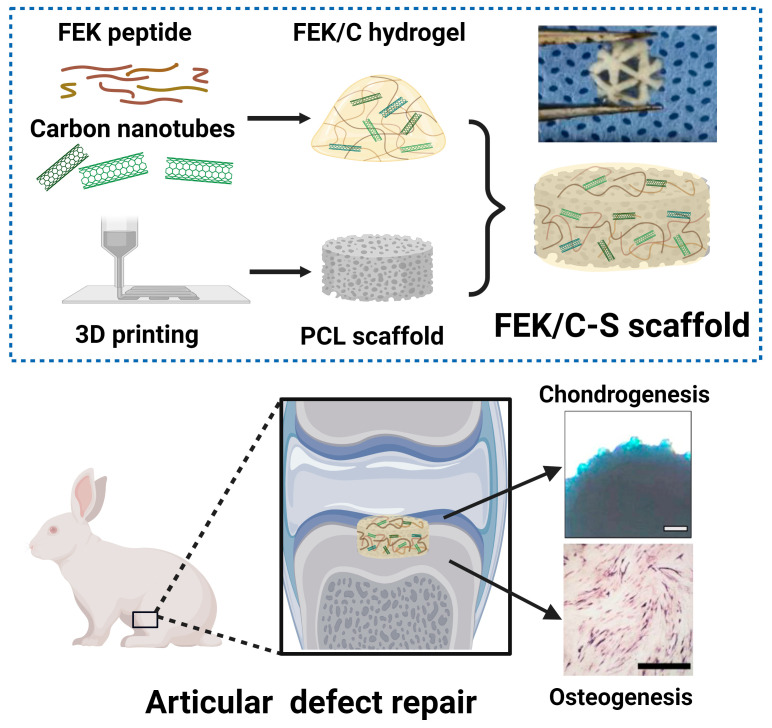
Schematic illustration of the fabrication of CNT-doped peptide hydrogel–PCL composite scaffolds and their application in articular defect repair. Scale bars: 25 μm (white) and 1 mm (black).

In this study, we developed a composite scaffold for cartilage defect repair by incorporating a CNT-doped octapeptide hydrogel onto a 3D-printed PCL scaffold (FEK/C-S), as illustrated in the Scheme 1. CNTs were fabricated in the network of the previously introduced non-immunogenic octapeptide (FEFEFKFK) to form a novel hydrogel denoted as FEK/C. Then, the FEK/C was applied to a 3D-printed PCL scaffold to form a composite scaffold denoted as FEK/C-S. The FEK/C-S demonstrated excellent biocompatibility and promoted the osteogenesis and chondrogenesis processes both in vitro and in a rabbit cartilage/subchondral bone defect model. 

## 2. Methodology

### 2.1. Materials

All chemicals were purchased from Aladdin and used as received if not specifically mentioned. Octapeptide with a sequence of FEFEFKFK was prepared in our lab and stored at −20 °C, as described in previously published papers [26]. Multi-wall carbon nanotubes (CNTs) were purchased from Chengdu Organic Chemistry Co., Ltd., Chinese Academy of Science, Chengdu, China. Simulated body fluid (SBF) was purchased from Shanghai yuanye Bio-Technology with a formula reported by Kokubo et al. [37]. The water used in the experiment was deionized water (Ω = 18.2 at ambient temperature) passing through a 0.22 μm filter before use.

### 2.2. Preparation of Octapeptide-CNT Hybrid Hydrogel (FEK/C) 

FEK/C hydrogel was prepared by mixing CNT and the FEFEFKFK octapeptide in a water solution, followed by pH adjustment. In brief, 5 mg/mL of CNTs were prepared by dissolving CNT powders in water and dispersing them with 30 min ultrasound treatment using a water bath (Sonic-1000WT, Newtown, CT, USA). The as-prepared CNT solution was mixed with octapeptide aqueous solution using vortex at dilutions of 1:10, 1:50, and 1:100 for FEK/C_1_, FEK/C_2_, and FEK/C_3_, followed by heating in an 80 °C oven for 3 h. After cooling down to ambient temperature, the pH of the mixture was adjusted to 7.2 ± 0.2 and the volume was topped up to make the final FEK concentration 20 mg/mL. Octapeptide hydrogel without CNT doping was also prepared using this method.

### 2.3. Preparation of FEK/C Composite PCL Scaffold

The PCL scaffold was printed using a fused deposition modelling 3D printer (Creality CR-2020, Shenzhen, China). In brief, the PCL was infilled into a syringe and melted at 95 °C for printing using 0°/60°/120° angles. The size of the PCL scaffolds (diameter × height) was altered in different experiments: 5 mm × 4 mm for characterization and in vivo implantation, 5 mm × 1 mm for the cellular experiment using a 96-well plate, and 33 mm × 1 mm for the cellular experiment using a 6-well plate.

To prepare FEK/C composite PCL scaffolds, the PCL scaffold was soaked into the as-prepared FEK/C hydrogels overnight at ambient temperature. For comparison, FEK hydrogel was applied to prepare a CNT-free scaffold (FEK-S). 

### 2.4. Characterizations

TEM observation: CNTs with different dispersion methods were diluted to a final concentration of 2 μg/mL and applied to a 300 mesh copper grid. The TEM images were taken using a Talos L120C TEM (Thermo Scientific, Waltham, MA, USA) under 120 kV voltage. The diameter of CNTs was measured using ImageJ software.

SEM observation: The hydrogels and scaffolds were lyophilized for 48 h for sample preparation. Then, each sample was gold-plated, and the morphology was observed using a JSM-6510 SEM (JEOL, Tokyo, Japan) with 5 kV voltage.

Raman spectrum: Lyophilized powders of FEK/C_1_, FEK/C_2_, and FEK/C_3_ were put on slices, respectively. The Raman spectra were detected under a 532 nm excitation wavelength. 

Circular dichroism: To prepare samples for circular dichroism detection, the CNT solution was diluted at 1:100, and FEK/C or FEK hydrogels were diluted at 1:60. The diluents were applied to a Chirascan circular dichroism spectrometer (Applied Photophysics, London, UK) for recording the spectra between 185 to 250 nm, respectively.

Oscillatory rheology: FEK and FEK/C solutions were prepared with a final FEK concentration of 20 mg/mL. For each sample, the pH was adjusted to 3.0 and 7.0, respectively. The energy storage modulus (G′), energy loss modulus (G″), and complex viscosity were recorded using an AR-G2 rheometer (TA instruments, New Castle, DE, USA) under 1% strain at ambient temperature.

Fourier transform infrared spectrum (FTIR): Lyophilized hydrogels and scaffolds were measured using a Fourier infrared spectrometer (Evolution 201, Thermo Scientific, USA). The samples were scanned with a resolution of 4. 

Water contact angle: The water contact angles were measured by imaging a water drop on the PCL scaffold or composite scaffolds using a static water contact angle meter (Maishi DropMeter A100P, Ningbo, China). Each scaffold was tested 3 times.

Conductivity: The resistance (R) of scaffolds was tested using a multimetre. Then, the conductivity (σ) was calculated using the following equation:

Ρ = RA/L, σ = 1/ρ,(1)

where ρ is for resistivity, A is for the area, and L is for the length.

PCL scaffolds soaking in deionized water for 12 h were used as control, and the conductivity of this control was tested to be 18.2 MΩ.cm at ambient temperature. All experiments were repeated three times.

Protein absorption: An aqueous solution of bovine serum albumin (BSA) was prepared with a concentration of 0.5 mg/mL. To test the protein absorption ability, different scaffolds (5 mm × 4 mm) were soaked in 1 mL of the BSA solution for 4–72 h. At each predetermined time point, 200 μL of supernatant was taken, and the protein concentration was tested using a BCA kit (Beyotime, Nantong, China). All experiments were repeated three times. The protein absorption for each scaffold was calculated using the following equation:

Absorbed protein (mg) = Initial protein (mg) − Protein in supernatant (mg).(2)

Haemolytic assay: Red blood cells (RBCs) were collected by centrifugation of fresh rabbit blood at 1500 rpm for 15 min, followed by dispersing in 0.9% NaCl solution to make a 5% RBC suspension. Then, 50 μL of RBC suspension was transferred into a 96-well plate with scaffolds containing 200 μL of 0.9% NaCl. Scaffold-free wells containing 200 μL of 0.9% NaCl or deionized water were used as negative and positive controls, respectively. After a 5 h incubation at 37 °C, the supernatants were collected, and RBCs were removed by centrifuge. A UV spectrophotometer (Evolution 201, Thermo Scientific, USA) was used to record the absorbance at 576 nm. All experiments were repeated three times. The haemolytic rate (%) was calculated using the following equation:

Haemolysis rate (%) = (A _sample_ − A _negative control_)/(A _positive control_ − A _negative control_) × 100%. (3)

In vitro dissolution observation: Scaffolds were soaked in simulated body fluid (SBF) at 37 °C for 30 days. Then the SEM images were taken to observe the morphology.

### 2.5. Cell Culture

Articular cartilage cells (ACs), osteoblasts (OBs), and bone marrow mesenchymal stem cells (BMSCs) were provided by Liming Wang’s lab from Nanjing First Hospital for laboratory use only [38]. All the cells were cultured in Dulbecco’s modified Eagle medium (DMEM) supplemented with 10% foetal bovine serum (FBS) and 1% penicillin/streptomycin. To prepare sterilized scaffolds for cell culture, the 3D-printed PCL scaffolds were soaked in 75% ethanol overnight, then rinsed with sterilized water and dried in a biosafety cabinet. FEK and CNT powders were treated with UV for 30 min. Then, the FEK/C hydrogel and FEK/C-S were fabricated under sterilized conditions. All experiments were performed with at least three biological repeats if not specifically mentioned. 

### 2.6. In Vitro Compatibility

The sterilized scaffolds were soaked in a culture medium in a 96-well plate overnight. Then, ACs, OBs, and BMSCs were seeded in the plate with a density of 5000 per well. After incubation with different scaffolds for 1, 3, and 5 days, the cell viability was tested using a CCK8 reagent (Keygentic, Nanjing, China) according to the manufactory’s protocol. Simultaneously, calcein AM/propidium iodide (PI) reagent (Keygenitic, Nanjing, China) was applied to the cells after incubating for 3 days with different scaffolds. The samples were observed using a fluorescence microscope (Shunyu, Yuyao, China) to visualize the live and dead cells. 

Giemsa staining was applied to visualize the morphology of ACs, OBs, and BMSCs. Cells were seeded in a 96-well plate with a density of 5000 cell per well. After a 3-day co-culture with different scaffolds, the medium with scaffolds were discarded, and each well was rinsed with PBS, fixed with 4% paraformaldehyde (PFA) for 20 min. The cells were stained with modified Giemsa staining solution (Beyotime, Nantong, China) for 30 min. After rinse against PBS, the specimens were visualized under microscope. 

### 2.7. In Vitro Migration and Adhesion Assessment

The scratch assay was applied to evaluate the in vitro migration ability of BMSCs. In brief, cells were plated in a 6-well plate at a density of 1 × 10^5^ cells/well. When they reached 90% confluency, scratches were made using a 200 μL pipette tip at the bottom of each well. After removing the detached cells, PCL or composite scaffolds were put into the wells containing 2 mL of serum-free medium. After a 24 h incubation period, the plate was imaged using a CX23 microscope (Olympus, Tokyo, Japan). The migration rate was calculated using Image J software (version 1.53). 

The AC adhesion to scaffolds was tested using a CCK8 kit. In brief, ACs were plated in a 96-well plate containing a pre-soaked PCL or FEK/C composite scaffold at a density of 1 × 10^4^ cells/well. After 2–8 h, the scaffolds were rinsed twice with PBS before transferring to a new plate and incubating with CCK8 reagent. The cell adhesion to the scaffolds was quantified by measuring the absorbance at 450 nm according to the manufactory’s protocol. 

### 2.8. Assessment of Osteogenesis 

BMSCs were seeded in a 6-well plate containing DMEM at a density of 2 × 10^4^ cells/well. When they reached 70% confluency, the medium was replaced by DMEM containing extra 1% glutamine, 0.2% ascorbic acid, 1% sodium β-glycerophosphate, and 0.01% dexamethasone. The BMSCs were cultured for another 7 or 14 days, then rinsed twice with PBS, and stained with an alkaline phosphatase (ALP) kit and an alizarin red reagent (only for the 14-day culture), respectively. All the staining was performed according to the manufacturer’s protocol of Beyotime (Nantong, China). The samples were then visualized using an optical microscope, and the results were quantified using Image J software (version 1.53).

### 2.9. Assessment of Chondrogenesis

PCL and FEK/C-S scaffolds were sterilized and soaked in DMEM in a 96-well plate before use. Then, ACs were seeded in the 96-well plate at a density of 2 × 10^4^ cells/well. After 7 or 14 days, the supernatant was collected for testing the glycosaminoglycan (GAG) concentration using an anti-rabbit GAG Elisa Kit (Huabang, Shanghai, China). The Elisa detection was performed according to the manufactory’s protocol.

For cartilage sphere culture, 50 μL of DMEM solution containing 1% agar was infilled in each well of a 96-well plate. After 2 h for cooling down, the ACs were seeded on the DMEM/agar matrix at a density of 2 × 10^4^ cells/well. The formation of a cartilage sphere was monitored using an optical microscope. When the cartilage sphere formed, 10 μL of FEK/C hydrogel was replenished into each well with care. Simultaneously, 10 μL of sterilized water was added as a control. After 14 days, the cartilage sphere was fixed with 4%PFA and stained with alcian blue for 30 min. The stained sphere was rinsed and visualized using an optical microscope. 

### 2.10. Animal Handling and Model Establishment

All the animal handling and surgical procedures were approved by the ethics committee of Nanjing First Hospital (DWSY-23041345). Six-month-old female New Zealand white rabbits were purchased from Hengtai Experimental Animal Breeding Co., Ltd, Wuxi, China. To establish a bone defect model, a hole (Φ6 mm × 4 mm) was drilled on the medial knee of each rabbit. The sterilized FEK/C-S or PCL scaffolds were implanted into the hole for repair before stapling. After surgery, 20 U of penicillin was injected intramuscularly every 3 days. The rabbits were sacrificed, and the knee joints were harvested for further analysis at 8 weeks post-surgery. 

### 2.11. Micro-CT Imaging and Bone Mass Analysis

The knee joints were scanned using a SkyScan 1176 Micro-CT machine (Bruker, Mannheim, Germany) at 70 KV voltage and 9 μm pixels, followed by reconstruction of 3D models. Bone mass analysis was carried out using a Bruker’s CTan software (version 1.6.10.1) to determine the bone volume/tissue volume (BV/TV), the bone mineralization density (BMD), the trabecular number (Tb.N), and the trabecular thickness (Tb.Th).

### 2.12. Histological Analysis

The knee joints were treated with 4% PFA and 10% EDTA. Then, the softened knee samples were embedded into paraffin and cut into 5 μm-thick specimens using a microtome. The specimens were stained according to the standard protocols for haematoxylin and eosin (H and E), safranine-O/fast green, and Masson staining. All the stained specimens were imaged, and typical images were presented according to the suggestion of histologists. 

### 2.13. Statistical Analysis

All the experiments were performed with at least 3 repeats if not specifically mentioned. The results were shown as mean ± S.D. Data analysis was performed using GraphPad Prism software (version 8.4.3). An unpaired two-tailed *t*-test was used to compare between two groups. One-way ANOVA with Tukey’s post-test was used to compare between multiple groups. Results were presented as mean ± SEM, with statistically significant signs (*, *p* < 0.05; **, *p* < 0.01; ***, *p* < 0.001, and ****, *p* < 0.0001). 

## 3. Results

### 3.1. Characterization of FEK/C Hydrogel

The bulk CNTs were subjected to different processing methods, namely vortexing, high-speed shearing, and sonication to form a stable aqueous solution. Among these methods, the CNT dispersion treated with sonication demonstrated superior stability, as depicted in Figure S1, even after a minimum of 30 min. TEM images, as shown in Figure S2, revealed that sonicated CNTs were uniformly dispersed with fewer entanglements when compared to CNTs processed with vortexing and high-speed shearing, which exhibited significant agglomeration. The distribution status of CNTs is directly linked to the stability of their dispersions. 

**Figure 1:**
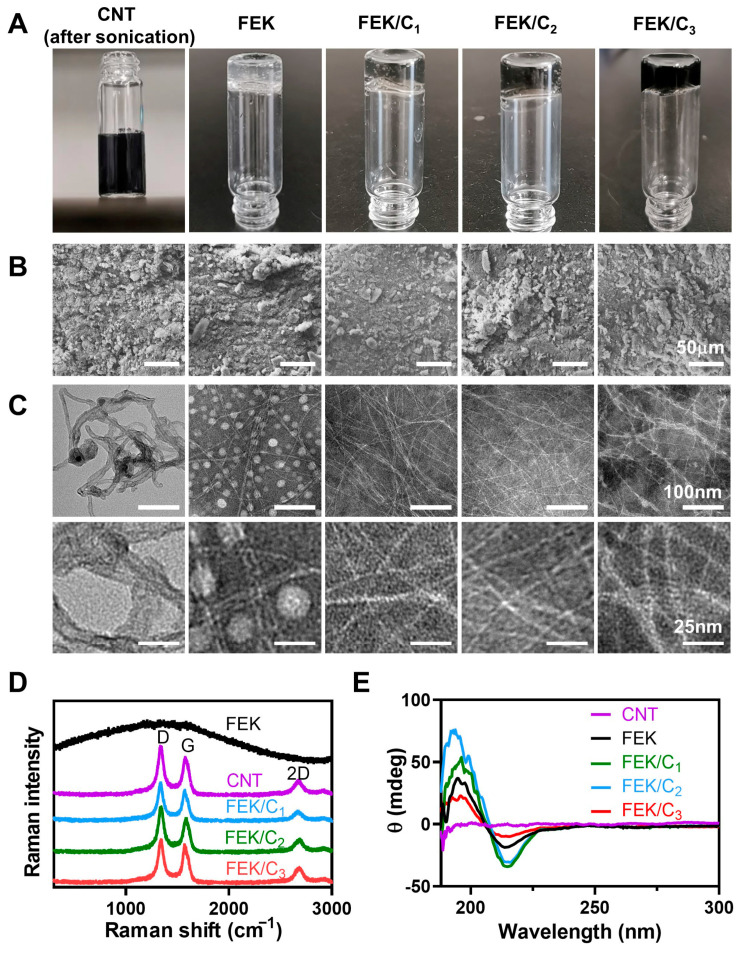
Characterization of CNT-doped FEK peptide hydrogels. (**A**) Images of CNT solution after sonication and the formed FEK or FEK/C hydrogels; (**B**) SEM images, (**C**) TEM images, (**D**) Raman spectrum, D/G/2D represent the corresponding characteristic peaks, respectively; and (**E**) circular dichroism chromatogram spectra of FEK-based hydrogels containing different concentration of CNTs.

The sonicated CNTs were incorporated into the octapeptide solution to create CNT-doped self-assembly peptide hydrogels (FEK/C). These hydrogels were labelled as FEK/C_1_, FEK/C_2_, and FEK/C_3_ based on the concentrations of CNTs used, while the pristine peptide hydrogel without CNT doping was referred to as FEK (Table 1). At ambient temperature, the octapeptide alone formed a semi-transparent hydrogel, as shown in Figure 1A. However, upon CNT doping, the hydrogels turned black in colour. Inverted images confirmed that all the hydrogels formed were non-liquid, indicating their stability. To examine the microstructure of the hydrogels with varying CNT doping concentrations, electron microscopes were employed. In Figure 1B, scanning electronic microscopy (SEM) images revealed stacked layers and bulk structures for FEK, consistent with previous reports [23]. The FEK/C_1_, FEK/C_2_, and FEK/C_3_ hydrogels exhibited similar morphologies to FEK under SEM. When stained with phosphotungstic acid, transmittance electronic microscopy (TEM) images demonstrated the entanglement of peptide fibres. Figure 1C illustrates that FEK displayed white fibre-like and sphere-like structures against a negatively stained background. However, after CNT doping, only fibre-like structures were observed, suggesting that CNTs facilitate fibre assembly and hydrogel network formation by enhancing interactions. The fibre network showed increased intersections with higher CNT concentrations, indicating that CNT doping may enhance the mechanical strength of the peptide hydrogels. Notably, the FEK/C_3_ hydrogel exhibited some fibre stacks and a disorganized network, indicating that an excessive amount of CNTs could hinder hydrogel formation. 

The infrared spectra comparison (Figure S3) between the CNT-doped hydrogels (FEK/C1, FEK/C2, and FEK/C3) and the pure FEK hydrogel reveals similar spectra, indicating successful doping of CNTs into the hydrogels without significant changes in their chemical composition. In addition, the FEK/C spectra did not show new peaks related to CNT when compared to the pure FEK spectrum. We postulated that this phenomenon may be caused by the low portion of CNT in the FEK/C, so the CNT-related peaks were too weak and covered by the strong peaks from the octapeptides. Nevertheless, the presence of CNT can be verified by other data, such as the dark colour morphology of hydrogels (Figure 1A), the microcosmic changes (Figure 1B,C), and the Raman spectra (Figure 1D). 

In the Raman spectra (Figure 1D), the characteristic peaks of pure CNTs (D and G peaks at 1350 cm^−1^ and 1580 cm^−1^, respectively, and a 2D peak at 2700 cm^−1^) are also observed in the spectra of FEK/C1, FEK/C2, and FEK/C3. This confirms the successful incorporation of CNTs into the hydrogels, suggesting that the CNTs maintained their structural properties within the hydrogel networks.

To assess the integrity and layers of the CNTs, the intensity ratios of G/D peaks (I_G_/I_D_) and 2D/G peaks (I_2D_/I_G_) were calculated [32]. According to Table 2, the CNT-doped hydrogels (FEK/C1, FEK/C2, and FEK/C3) exhibited I_G_/I_D_ values of 0.8–0.9 and I_2D_/I_G_ values around 0.43, which closely resemble the values of pure CNTs (0.45 in the table). These results indicate that the CNTs maintained their integrity and multi-layer properties within the formed hydrogel networks.

In the circular dichroism chromatogram (Figure 1E), the FEK shows a peak at 195 nm and a converted peak at 215 nm, suggesting the assembly of the FEK peptides into β-sheet stacks. With an increase in the CNT portion from 0 to 100 μg/mL, the intensities of these two peaks enhance, indicating enhanced β-sheet formation. However, FEK/C3 exhibits peaks with the weakest intensities, suggesting that an excessive amount of CNTs hinders the assembly of the octapeptide into β-sheet structures. These findings align with the observations from TEM, further confirming the influence of CNTs on the assembly of the hydrogel.

**Figure 2:**
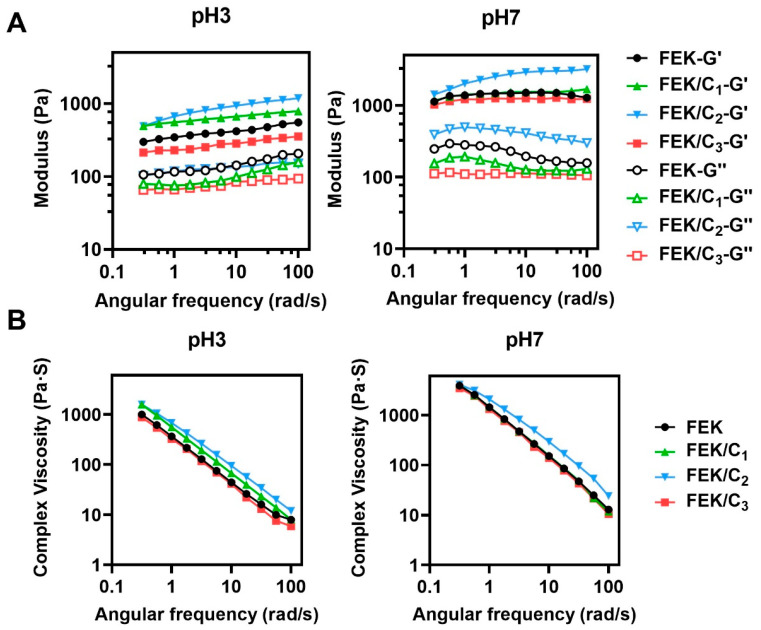
Mechanical properties of CNT-doped FEK peptide hydrogels. (**A**) The rheological modulus of hydrogels at pH 3 and 7; and (**B**) the complex viscosity with angular frequency at pH 3 and 7.

As in Figure 2A,B, the oscillatory rheological characteristics of the CNT-doped octapeptide were depicted under the solution form (at pH 3) and hydrogel form (at pH 7). At the different concentrations, FEK/C_2_ exhibited the highest elastic/storage modulus and the strongest complex viscosity across a range of angular frequencies at both pH 3 and pH 7. This indicates that FEK/C2 exhibits superior mechanical properties compared to the other hydrogel formulations. On the other hand, the modulus of FEK/C3 was lower than that of FEK, suggesting weaker mechanical performance in this particular formulation.

The results indicated that the performance of the hydrogels was enhanced by increasing the CNT concentration from 0 to 100 μg/mL. However, when the CNT concentration further increased to 500 μg/mL, the hydrogel performance was hindered. 

Theoretical considerations suggested that CNTs can assemble into the network of octapeptides by forming π–π stacks with the benzyl groups of phenylalanine residues. However, it is postulated that the CNT-octapeptide network may not be as stable as the pure octapeptide networks. The presence of CNTs could potentially interrupt hydrogen bond formation within the peptides or introduce additional hydrophobicity to the overall structure of the hydrogel. These factors may contribute to the observed differences in mechanical performance between the CNT-doped hydrogels and the pure octapeptide network.

### 3.2. Characterization of FEK/C-S Scaffold

**Figure 3:**
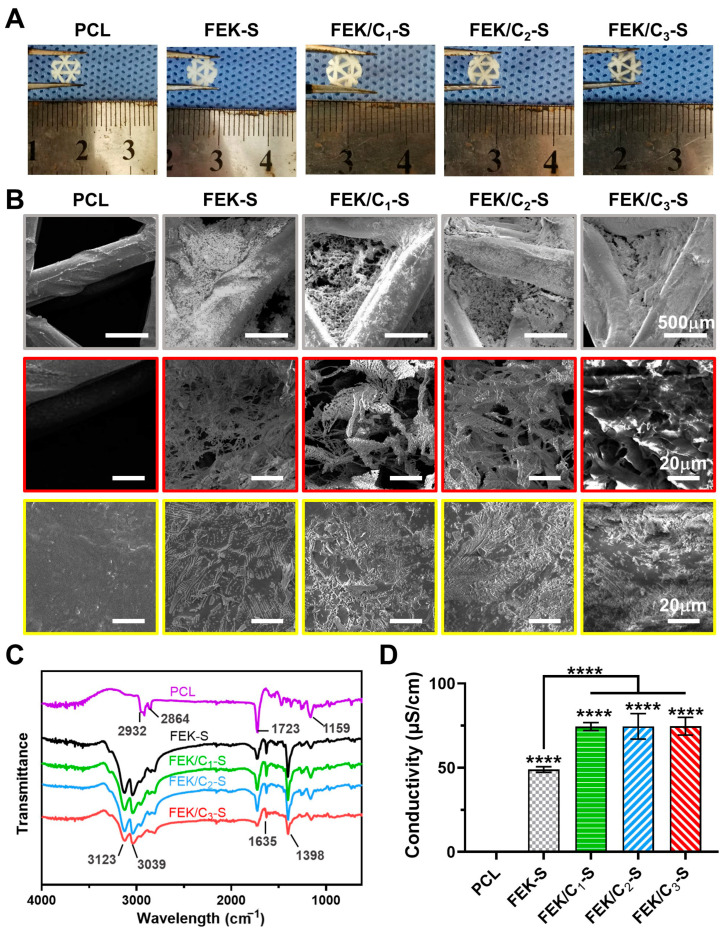
Characterization of FEK/C hydrogel–PCL composite scaffolds. (**A**) Photographs of PCL and hydrogel–PCL composite scaffolds; (**B**) SEM images of scaffolds, with typical areas of hydrogels infilled in the scaffold skeletons (in red frames) and on the PCL surfaces (in yellow frames) are shown below, respectively; (**C**) FTIR spectra and (**D**) conductivities of PCL and hydrogel–PCL composite scaffolds. The “****” above the column represents the statistical significance of this group compared to the PCL.

PCL was utilized to fabricate scaffolds using 3D printing technologies. As in Figure 3A, it can be observed that all the hydrogels were uniformly distributed around PCL to form composite scaffolds. Figure 3B depicted the scanning electronic microscopy observation, which showed that the octapeptide hydrogels were not only coated onto the PCL skeleton but also embedded within it. The microstructure of peptide hydrogel exhibited a porous morphology with crosslinked fibres. As the concentration of CNTs increased, the fibrous peptide overlapped and formed layered stacks within the scaffolds. 

As in Figure 3C, the PCL showed peaks at 2932, 2864, 1723, and 1159 cm^−1^, corresponding to the –CH_2_, –C=O–, –C–O–C– in the PCL backbone, respectively. With the FEK or FEK/C, new peaks were shown at 3123, 3039, 1635, and 1398 cm^−1^, corresponding to the –NH_2_, –NH–, and –C–H– groups of hydrogels. In general, these results suggested that the hydrogel and PCL scaffold were physical combined, rather than undergoing any chemical interactions. Due to the weak physical interaction forces, the composite hydrogel could degrade via (1) the leak out of the CNT from the hydrogel network and (2) the decomposition or digestion of the octapeptide in the physiological environment if incubated for a long time. These degradations may benefit bone repair by providing both spaces and nutrients for tissue formation around the PCL scaffold. It is worth noting that the pristine PCL scaffold is electrically insulated. However, with the incorporation of the FEK hydrogel, the conductivity increased from 0.2 to 49 μS/cm (Figure 3D). Furthermore, the CNT-doped composite scaffolds displayed even higher conductivity, reaching 74 μS/cm. Interestingly, within the tested range (0.05–0.5 mg/mL), there were no significant differences in conductivity observed among the scaffolds containing different CNT concentrations. Theoretically, the CNTs may keep shaping the electrochemical microenvironment even if they leaked out from the scaffold/hydrogel for a certain period, providing prolonged stimulation to facilitate bone repair.

**Figure 4:**
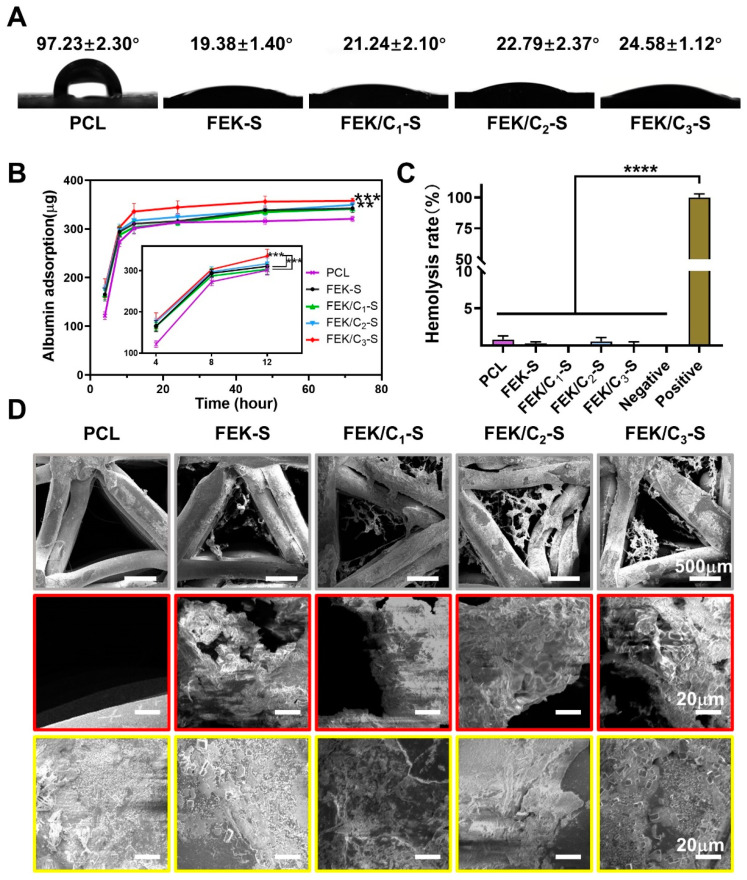
Effects of CNT-doped peptide hydrogel on the biocompatibility and degradation of composite scaffolds. (**A**) Contact angles of water on the surface of scaffolds; (**B**) Protein absorption on scaffolds from 4 to 72 h; (**C**) Haemolysis rate of rabbit red blood cells incubated with scaffolds for 5 h. The “****” represents the statistical significance compared to the Positive; (**D**) The degradation of scaffolds in simulated body fluids for 30 days, with typical areas of infilled hydrogels (in red frames) and PCL surfaces (in yellow frames) shown below, respectively. Negative: negative control that incubated with 0.9% NaCl; Positive: positive control that incubated with deionized water. The “**”and “***” at the right end of each line represents the statistical significance of this group compared to the PCL.

The physiological properties of the composite scaffolds were further evaluated in the study. In Figure 4A, it can be observed that the pristine PCL scaffold exhibited hydrophobic behaviour, as indicated by a water contact angle of 97.2°. On the other hand, the composite scaffolds showed improved hydrophilicity, with water contact angles ranging from 19 to 25°. The hydrophilicity slightly decreased with CNT increase in the FEK/C due to the intrinsic hydrophobicity of CNT [33]. In general, the FEK/C composition would be beneficial for cell attachment and growth on the scaffolds.

The protein absorption assay suggested that the absorption of albumin was low in the PCL group, whereas the FEK/C_3_-S and FEK/C_2_-S showed significantly higher absorption than the PCL group at 72 h (Figure 4B). Notably, the FEK/C_3_-S also showed higher absorption than FEK/C_1_-S and FEK-S at the initial 4 h. Its high absorption may be associated with the interaction of albumin and CNTs. As reported, the CNTs are able to adsorb a wide range of proteins and form a “protein corona” due to their physical/chemical characteristics at the interfaces [39,40]. In addition, the improved hydrophilicity of composite scaffolds may also be attributed to the higher protein absorption of all the composite scaffolds when compared to that of the PCL. This enhanced protein absorption suggests the potential for enhanced cell adhesion and biological activity on the composite scaffolds.

Furthermore, the haemocompatibility of the scaffolds was evaluated. As in Figure 4C, all the scaffolds exhibited a haemolysis rate lower than 5% after incubation with red blood cells for 5 h. This indicates good compatibility of the scaffolds with blood, suggesting their suitability for biomedical applications.

The degradation of the scaffolds was also investigated using SEM (JSM-6510, JEOL, Tokyo, Japan). Figure 4D showed the results after soaking the scaffolds in simulated body fluids (SBFs) for 30 days. The PCL network maintained its structure during the degradation process, with the formation of some crystals on the surface. However, the embedded hydrogels underwent significant shrinkage and agglomeration, resulting in the appearance of layered stacks. Among the three CNT-doped scaffolds, FEK/C2-S exhibited the highest preservation of the hydrogel component. This suggests that FEK/C2-S may have potential as a long-term implant due to its improved hydrogel retention during degradation.

### 3.3. Effects of FEK/C-S Scaffold on Cellular Biocompatibility 

**Figure 5:**
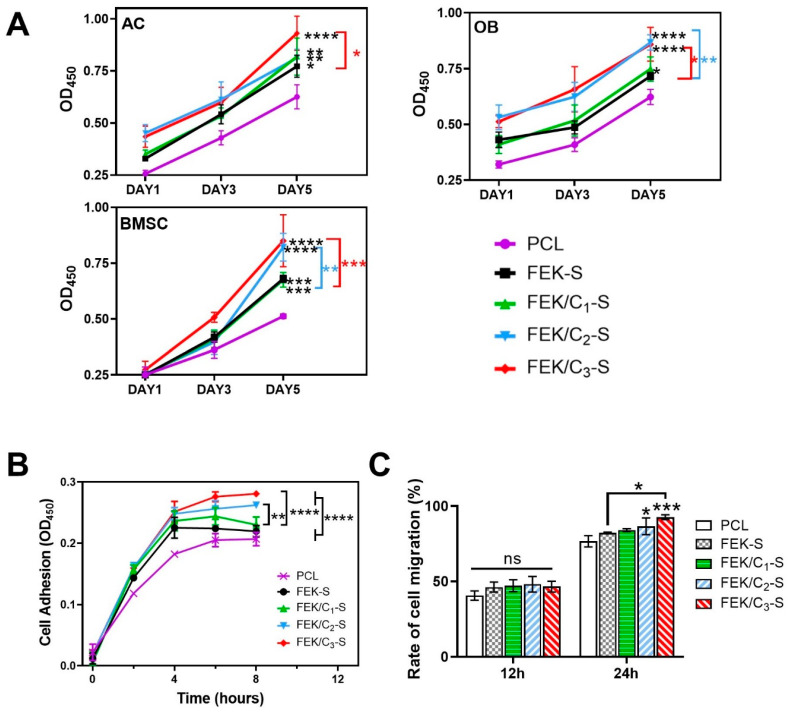
Effect of composite scaffolds on cellular biocompatibility. (**A**) The optical density values at 450 nm (OD_450_) of ACs, OBs, and BMSCs incubated with scaffolds for 1–5 days; (**B**) The OD_450_ values of ACs adhere on scaffolds after co-culture for 2–8 h; (**C**) The in vitro migration rate of BMSCs cultured with scaffolds. The “ns” refers to no significant differences between groups. The “*/**/***/****” at the right end of each line represents the statistical significance of this group compared to the PCL.

The biocompatibility of FEK/C-S scaffolds was assessed by co-culturing them with articular cartilage cells (ACs), osteoblasts (OBs), and bone marrow mesenchymal stem cells (BMSCs), respectively. In Figure 5A, all three cells can proliferate when co-cultured with scaffolds. Compared to the PCL scaffold, hydrogel-loaded scaffolds (FEK-S and FEK/C-S) exerted higher optical density (OD) values, suggesting enhanced cell proliferation on the composite scaffolds. Notably, the FEK/C2-S and FEK/C3-S scaffolds supported higher cell numbers compared to FEK-S on day five, suggesting that the incorporation of CNTs in the peptide hydrogel improved the microenvironment by enhancing the mechanical support, conductivity, and compactness of the hydrogel network.

Giemsa staining and live/dead staining images revealed that ACs, OBs, and BMSCs maintained normal morphologies when cultured with the scaffolds, indicating their compatibility with the cell types used (Figures S4 and S5). Due to the limited proliferation ability of ACs and OBs, bone repair relies on (1) the migration of BMSCs into the scaffolds, (2) the differentiated of BMSCs, and (3) the adhesion and accumulation of cartilage cells and osteoblasts on the scaffolds. To investigate the scaffold effect on cartilage repair, the cell adhesion ability to the scaffolds was evaluated using ACs (Figure 5B). The number of adhered ACs increased with the increase in CNT concentration. It has been reported that CNTs possess a high affinity for ECM proteins, such as collagen and fibronectin, which play important roles in cell adhesion [41,42,43]. Consequently, the incorporation of CNTs in the hydrogels improved AC adhesion to the scaffolds.

The in vitro migration ability was then tested (Figure 5C). In comparison to those with the PCL scaffold (76.6%) and the FEK-S (82.1%), BMSCs co-cultured with FEK/C_3_-S enhanced the migration rate (92.6%) at 24 h. Previous studies suggested that CNTs can regulate cell migration through an integrin-mediated mechanism [43,44]. These results were consistent with pioneering investigations on the effects of CNTs on cell migration.

### 3.4. Evaluation of Scaffold-Enabled Osteogenesis and Chondrogenesis

To assess the osteogenesis and chondrogenesis abilities of the scaffolds, we conducted several experiments.

**Figure 6:**
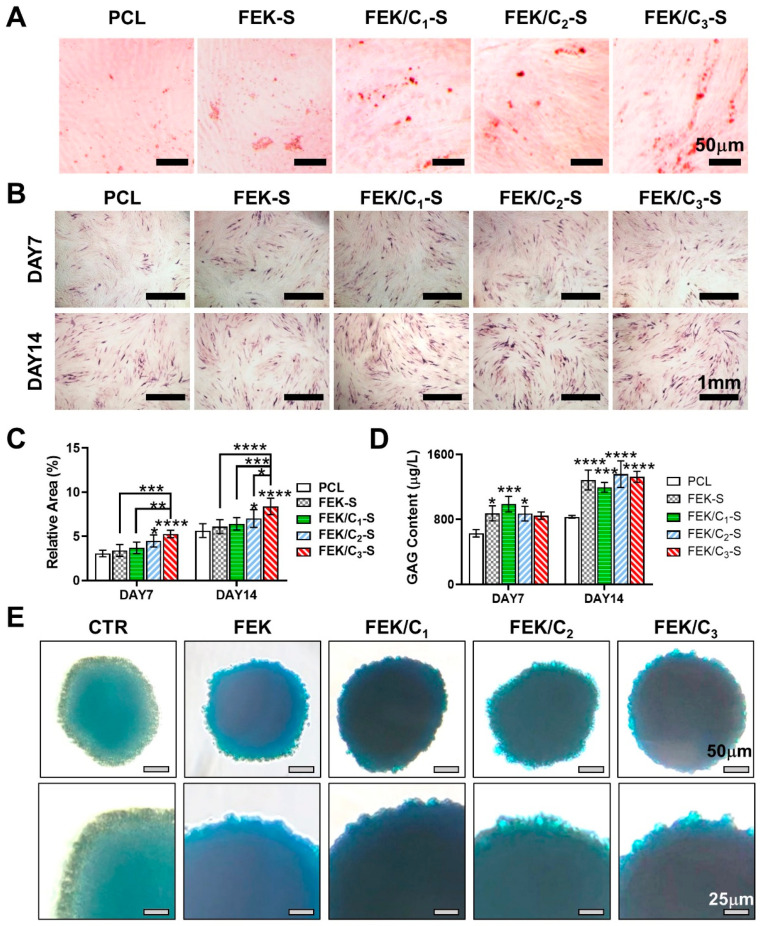
Effects of composite scaffolds on osteogenesis and chondrogenesis in vitro. (**A**) Alizarin red staining of BMSCs cultured with scaffolds for 14 days and (**B**) ALP staining of BMSCs for 7- and 14-day cultures; (**C**) The histogram diagram of ALP positive areas; (**D**) Quantification analysis of GAG secretion in the supernatant of BMSCs cultured with scaffolds; (**E**) The images of AC spheres stained with alcian blue, with high magnification images shown in the second row. The “*/**/***/****” above the column represents the statistical significance of this group as compared to the PCL.

In Figure 6A, red-stained calcium nodules were observed when BMSCs were cultured with scaffolds for 14 days, indicating the formation of calcium deposits and osteogenic differentiation. Among all the tested scaffolds, FEK/C_3_-S exhibited the highest number of calcium nodules, suggesting its ability to accelerate calcification.

We further evaluated the alkaline phosphatase (ALP) activity in scaffold co-cultured BMSCs by staining with para-nitrophenyl phosphate. As shown in Figure 6B,C, the hydrogel-loaded scaffolds exhibited higher levels of ALP compared to the pristine PCL scaffold. The ALP activity increased with the concentration of CNTs in the hydrogel. These results indicate that CNTs can promote the osteogenic differentiation of BMSCs in a dose-dependent manner. In Figure 6A,B, osteoblast-like cells were observed evenly distributed on all the scaffolds, indicating the good adhesion ability of osteoblasts and their potential application in subcartilage bone repair.

Sequentially, the chondrogenesis was quantified by measuring glycosaminoglycan (GAG) secretion in the culture medium. As depicted in Figure 6D, BMSCs cultured with the hydrogel-loaded scaffolds produced higher levels of GAGs when compared to those cultured with the PCL scaffold. The incorporation of CNTs in the hydrogel did not compromise the ability of the scaffolds to support high GAG formation. 

To maintain the phenotype of ACs, which typically undergo phenotype loss during in vitro cell culture, an AC sphere culture approach was established to mimic the in vivo microenvironment. In Figure 6E, the inner layers of AC spheres stained blue under all culture conditions, indicating the formation of cartilage phenotype-related acidic polysaccharides. Notably, the outer layers of the AC sphere cultured on FEK/C_1_-S, FEK/C_2_-S, and FEK/C_3_-S were darker than those cultured on PCL and FEK-S, suggesting the hydrogels containing 0.05–0.5 mg/mL CNT facilitated the ECM sedimentation and phenotype maintaining for chondrocytes. This phenomenon may be due to (1) the advantageous landscape and mechanical support provided by the FEK/C hydrogels [29] and (2) the induction of local differentiation factors by π–π stacks.

### 3.5. Evaluation of Bone Repair Using FEK/C-S Scaffold 

**Figure 7:**
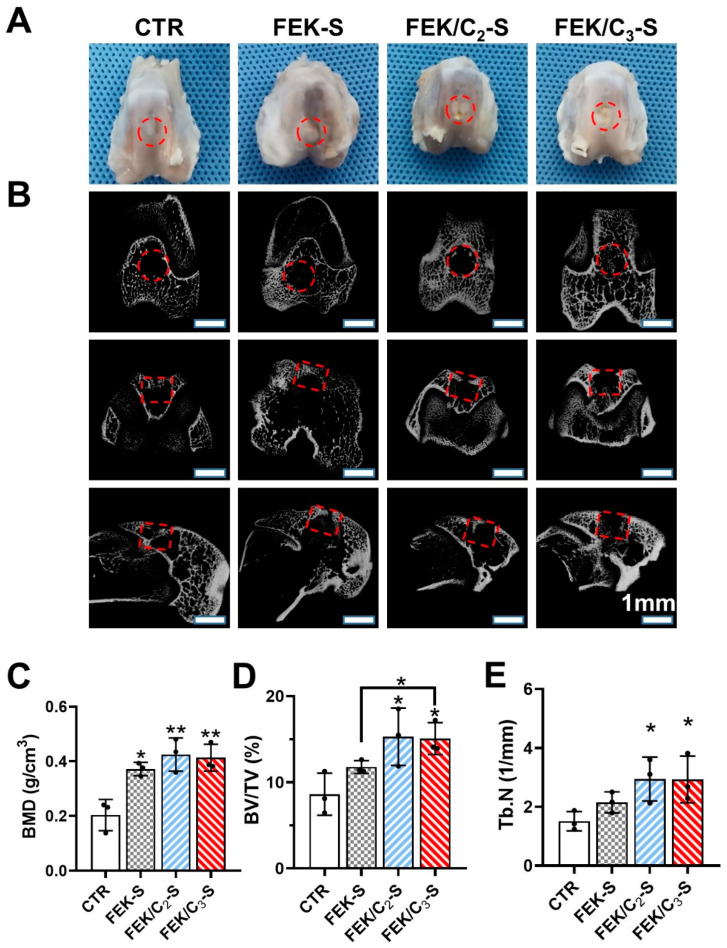
Effects of composite scaffolds on articular cartilage and subchondral bone defect repair in rabbits (**A**) Images of rabbit knee joints after articular defect and scaffold implant 8 weeks post-surgery; (**B**) Micro-CT images of repaired knee joint from coronal central plane (upper panel), transverse central plane (middle panel), and sagittal central plane (lower panel). The defect area is shown in a red circle (top view) or a red square (side view); (**C**) Bone mass density, (**D**) bone volume/tissue volume, and (**E**) trabeculae numbers calculated from Micro-CT. The “*/**” above the column represents the statistical significance of this group compared to the PCL.

To evaluate the composite scaffold-enabled cartilage repair, we established a knee joint defect model on rabbits (Figure S6). Due to the similar behaviours of FEK-S and FEK/C_1_-S in material characterization and cellular tests, only the FEK/C_2_-S and FEK/C_3_-S were involved in the animal work to investigate the influence of CNT-doping on bone repair. After 8 weeks, the knee joints of rabbits were covered with white chondroid tissues (Figure 7A). Some depressed areas can be observed in the scaffold-free (CTR) group, suggesting the uncompleted bone repair. The micro-CT reconstruction images confirmed a vacant part in the centre site (Figure 7B). The subchondral bone of the CTR and FEK-S groups was less than that of the FEK/C_2_-S and FEK/C_3_-S groups, distributing more around the cartilage and less in the defect centre (marrow cavity). In contrast, the FEK/C_2_-S and FEK/C_3_-S groups exhibited more hollowed bone tissues distributed around scaffolds, specifically some new bones close to the marrow cavity. As in Figure 7C,D, the statistical analysis revealed that the bone mineral density (BMD) and bone volume/tissue volume (BV/TV) showed similar trends as FEK/C_3_-S ≈ FEK/C_2_-S > FEK-S > CTR. These histomorphological parameters were in accordance with the observed trend of new bone formation. In addition, more trabeculae numbers (Tb.N) can be counted in the FEK/C_3_-S and FEK/C_2_-S groups than in the CTR group (Figure 7E). These data profiled that the new bone formed with FEK/C_2_-S and FEK/C_3_-S exhibited a hollowed structure like natural bone, and the scaffold implantation did not cause osteoporotic lesions.

**Figure 8:**
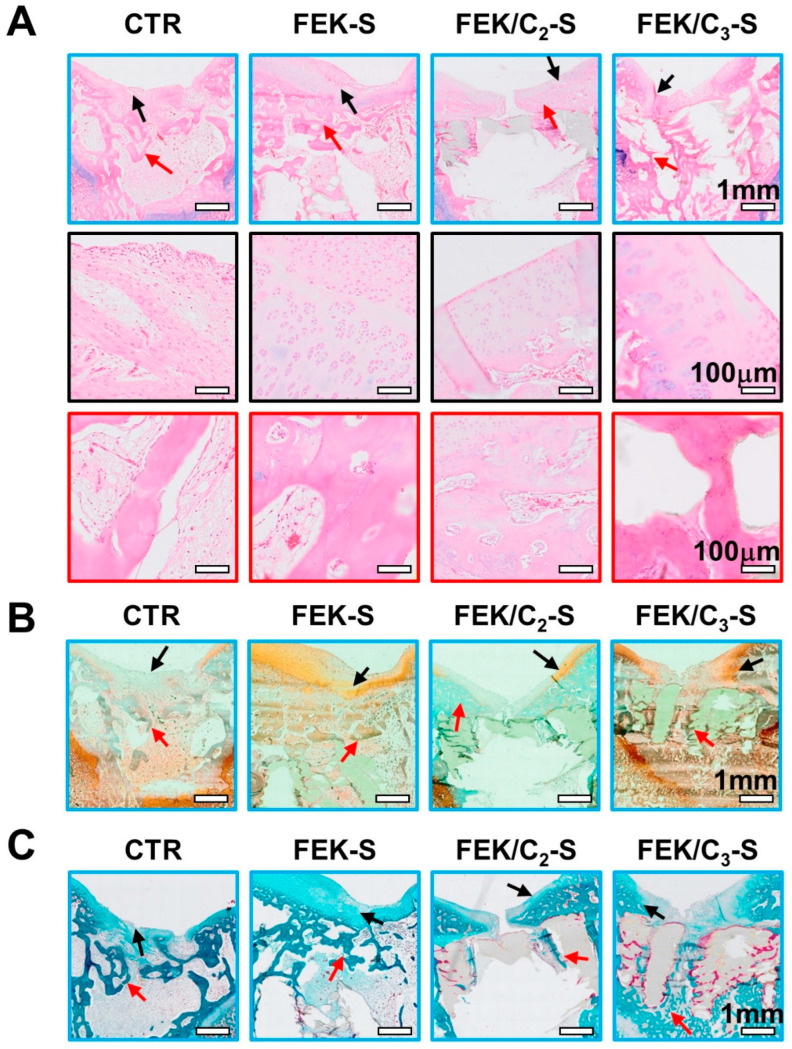
Histological analysis of rabbit knee joints. (**A**) H and E staining of the knee joint (first row) with high magnification of the cartilage (second row) and subchondral bone (third row); (**B**) Safranin-O/fast green staining and (**C**) Masson staining of the defect joint after being treated with different scaffold for 8 weeks. Black and red arrows indicated cartilage and subchondral bone, respectively.

Histological staining was performed to evaluate the regeneration of cartilage and subchondral bone in the knee joint defect model. Haematoxylin and eosin (HE) staining images revealed loose fibrous tissue filling the cartilage defect site in the CTR group, with some trabecular structures observed in the subchondral area (Figure 8A). However, most of the defect area was filled with pathological fibrosis. No significant inflammation was observed in any of the scaffold-implanted samples, indicating good biocompatibility of the hydrogel composite scaffolds.

Safranin-O/fast green staining was used to visualize the cartilage and subchondral bone. The FEK/C3-S group exhibited the most cartilage and subchondral bone formation among the four groups (Figure 8B). Enlarged views of the orange-stained area showed significant chondrocytes (Figure S7). The CTR group had less orange and green staining compared to the scaffold groups, indicating that the hydrogels provided an ECM-mimetic microenvironment for cell attachment and cartilage matrix deposition.

Masson staining was employed to illustrate the new bone formation, represented by the red-stained area. In Figure 8C, the FEK/C_3_-S group showed evenly distributed blue stains in the defect area, consistent with the micro-CT observations. A red-stained new bone area was observed near the subchondral site of the FEK/C_3_-S group, suggesting accelerated osteogenesis within this 0.5 mg/mL CNT-doped FEK hydrogel composite scaffold network compared to the other groups (Figure S8). 

The FEK/C3-S scaffold promoted cartilage and subchondral bone repair in the rabbit joint defect model. Histological staining confirmed the formation of new tissue, including cartilage and subchondral bone, and demonstrated the regenerative potential of the CNT-doped hydrogel composite scaffold.

## 4. Conclusions

In conclusion, the developed CNT-doped FEK peptide self-assembled hydrogel and the hydrogel-based PCL composite scaffold show promising results for cartilage and bone repair applications. By adjusting the CNT concentration, the hydrogel’s properties, such as microstructure, mechanical characteristics, and conductivity, can be modulated. The composite scaffold, with an optimal CNT doping level, promotes osteogenesis and chondrogenesis of BMSCs, enhances cell migration and adhesion, and provides a favourable microenvironment for tissue regeneration. Implantation of the FEK/C3-S composite scaffold in a rabbit knee joint defect model resulted in accelerated regeneration of cartilage and subchondral bone 8 weeks post-surgery. These findings highlight the potential of CNT-doped hydrogel–PCL composite scaffolds for effective cartilage and subchondral bone repair in real-life scenarios.

## Figures and Tables

**Table 1:** The nomenclature of peptide, hydrogel, and scaffolds.

Name	Content
Octapeptide	A synthesized peptide with a sequence of FEFEFKFK
FEK	Self-assembled peptide hydrogel
FEK/C_1_	Self-assembled peptide hydrogel containing 0.05 mg/mL CNT
FEK/C_2_	Self-assembled peptide hydrogel containing 0.1 mg/mL CNT
FEK/C_3_	Self-assembled peptide hydrogel containing 0.5 mg/mL CNT
FEK-S	PCL scaffold composited with FEK
FEK/C_1_-S	PCL scaffold composited with FEK/C_1_
FEK/C_2_-S	PCL scaffold composited with FEK/C_2_
FEK/C_3_-S	PCL scaffold composited with FEK/C_3_

**Table 2:** The intensity of D, G, and 2D peaks from CNT and hydrogels in Raman spectra.

Content	I_G_	I_D_	I_2D_	I_G_/I_D_	I_2D_/I_G_
CNT	371.25	453.75	168.75	0.82	0.45
FEK/C_1_	302.5	366.25	122.5	0.83	0.40
FEK/C_2_	323.75	421.25	140	0.77	0.43
FEK/C_3_	363.75	405	165	0.90	0.45

## Data Availability

Raw data and analysis files generated during the study are available from the corresponding author (Zhu Y.) on reasonable request.

## Supplementary Materials

The following supporting information can be downloaded at: https://www.mdpi.com/article/10.3390/pharmaceutics15082145/s1, Figure S1. Images of CNT dispersions prepared by different processing methods. Figure S2. TEM images of CNTs with different processing methods. Figure S3. FITR spectrum of hydrogels with different CNT concentrations. Figure S4. Giemsa staining of ACs, OBs, and BMSCs cultured on different scaffolds. Figure S5. Livedead staining of ACs, OBs, and BMSCs cultured on different scaffolds. Figure S6. Typical images of (left) a rabbit knee with cartilage and subchondral bone defect and (right) scaffold implantation during the surgery. Figure S7. High magnification images of (a–d) cartilage and (e–h) subchondral bone with safranin-O/fast green staining. Figure S8. High magnification images of (a–d) cartilage and (e–h) subchondral bone with Masson staining.
