# Supplementary material for: Enhanced Cartilage and Subchondral Bone Repair Using Carbon Nanotube-Doped Peptide Hydrogel–Polycaprolactone Composite Scaffolds
Source: Pharmaceutics. 2023 Aug 15;15(8):2145. doi: 10.3390/pharmaceutics15082145 (PMC10458387; doi:10.3390/pharmaceutics15082145)
Supplement: Supplementary file 1 [file pharmaceutics-15-02145-s001.zip › pharmaceutics-2518471-supplementary.pdf]

Enhanced Cartilage and Subchondral Bone Repair using Carbon Nanotube-doped Peptide Hydrogel-Polycaprolactone Composite Scaffolds

Jiayi Lv[a], Yilun Wu[a], Zhicheng Cao[b], Xu Liu[a], Yuzhi Sun[b], Po Zhang[b], Xin Zhang[b], Kexin Tang[c], Min Cheng[a], Qingqiang Yao[b*], Yishen Zhu[a*]

*Corresponding authors

a. College of Biotechnology and Pharmaceutical Engineering, Nanjing Tech University, 211816, Nanjing, China

b. Department of Orthopaedic Surgery, Institute of Digital Medicine, Nanjing First Hospital, Nanjing Medical University, 210006, Nanjing, China

c. College of Pharmaceutical Sciences, Nanjing Tech University, 211816, Nanjing, China

E-mail: yaoqingqiang@njmu.edu.cn (Q.Y.); zhuyish@njtech.edu.cn (Y.Z.);

Tel: +86-18900660563 (Y.Z.)

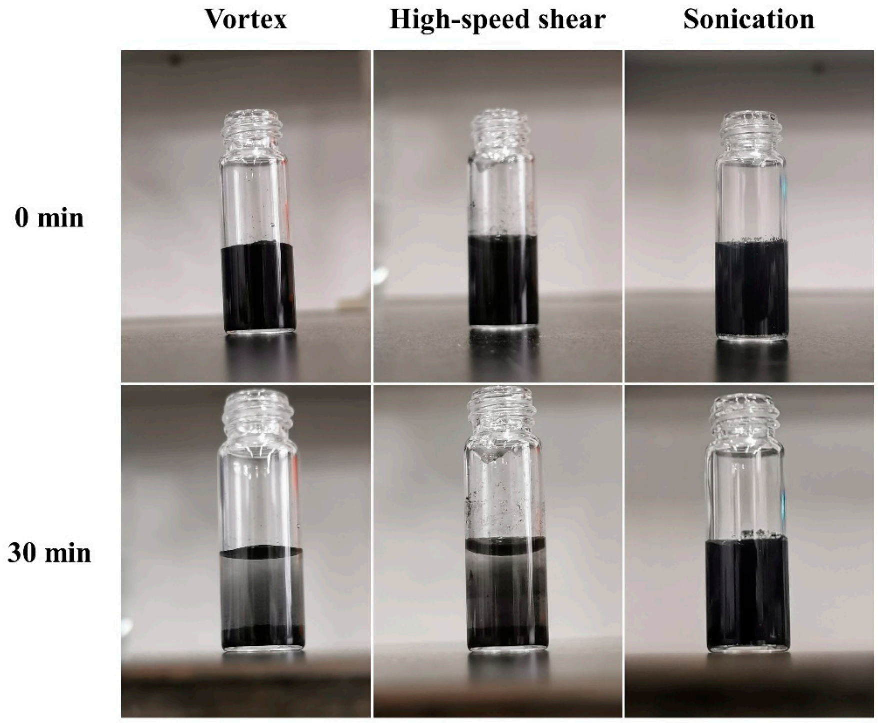

Figure S1. Images of CNT dispersions prepared by different processing methods.

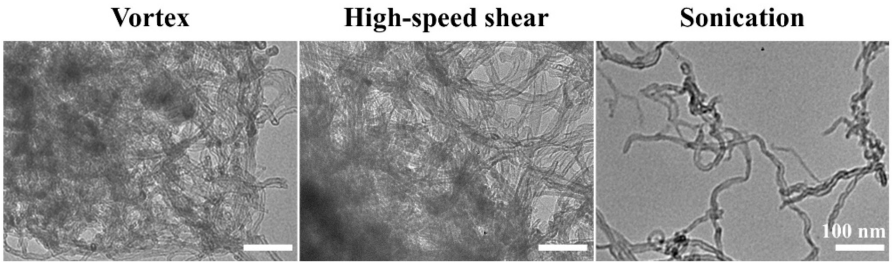

Figure S2. TEM images of CNTs with different processing methods.

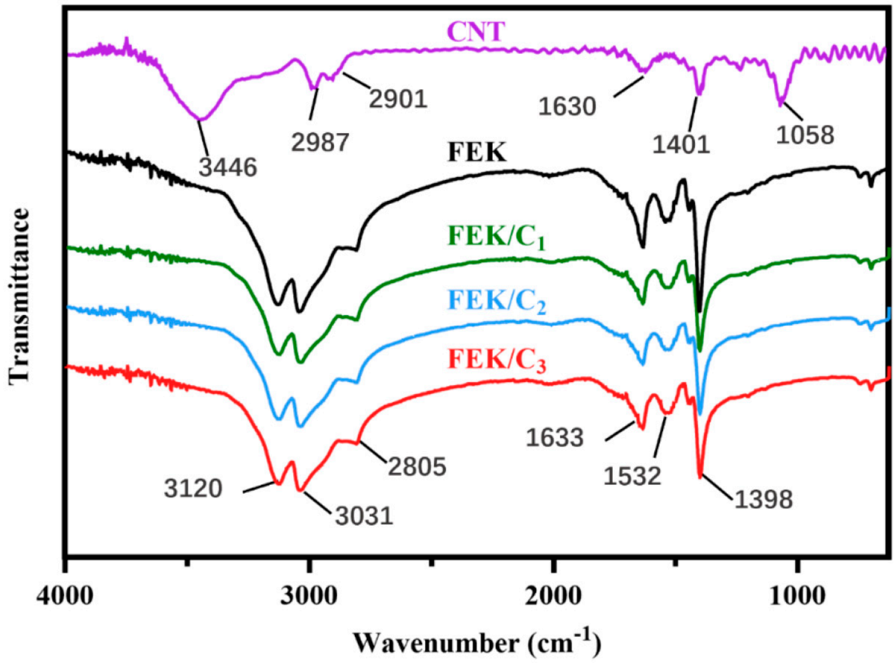

Figure S3. FITR spectrum of hydrogels with different CNT concentrations. The corresponding peaks of CNT: O-H stretching vibration peak (3446cm^{-1}) from H_2O, $-CH_2-$ stretching peak (2987 and 2901 cm^{-1}) and C-C stretching vibration peak (1401 cm^{-1}) from the backbone of CNT, C=O stretching vibration peak (1630 cm^{-1}) and C-O stretching vibration peak (1058 cm^{-1}) from oxidative CNT. The peaks in the FEK/C spectra: $-NH_2$ stretching vibration peak (3120 and 3031 cm^{-1}), $-CH_2-$ stretching vibration peak (2805 cm^{-1}), -NH- bending vibration peak (1633 cm^{-1}), and -C-H- bending vibration peak (1532 cm^{-1}) from FEK octapeptide.

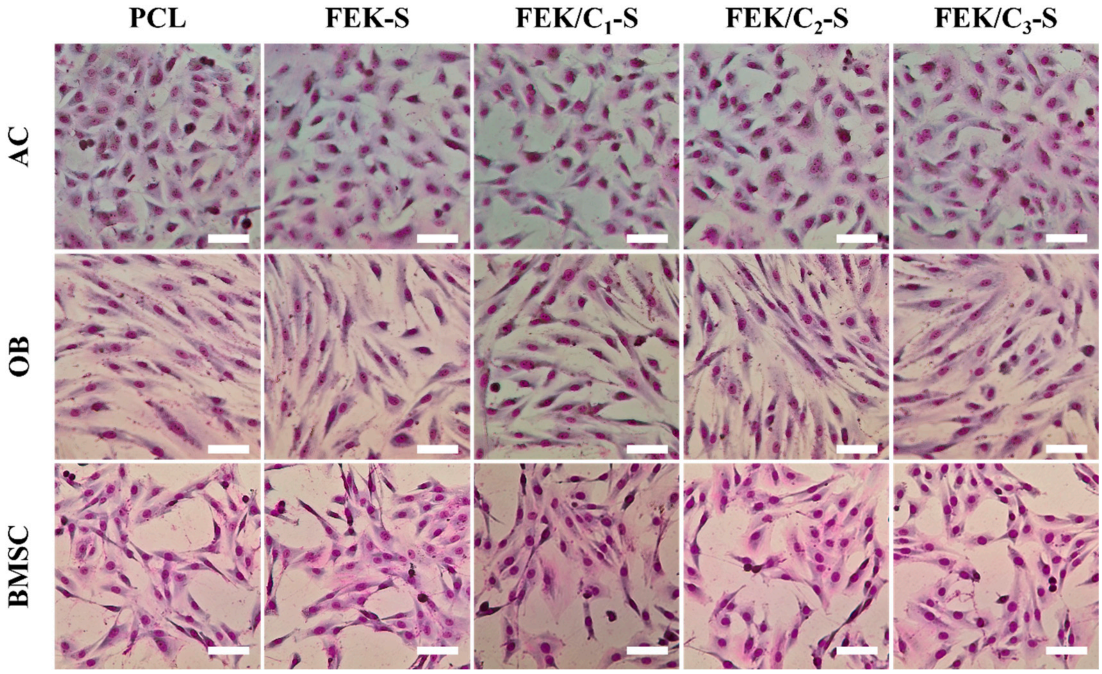

Figure S4. Giemsa staining of ACs, OBs, and BMSCs cultured on different scaffolds. Scale bar: 50 μm.

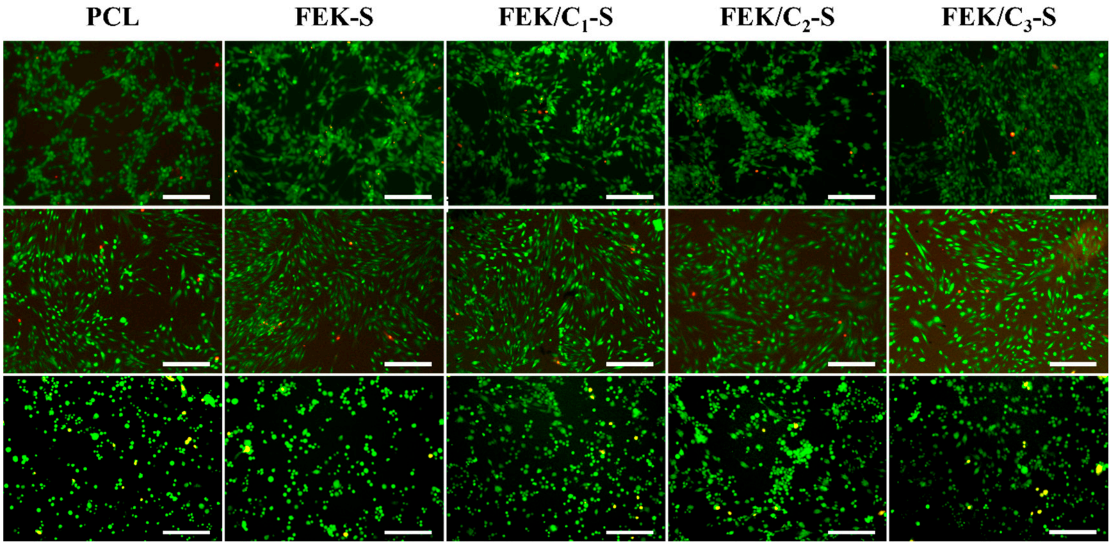

Figure S5. Livedead staining of ACs, OBs, and BMSCs cultured on different scaffolds. Scale bar: 200 μm.

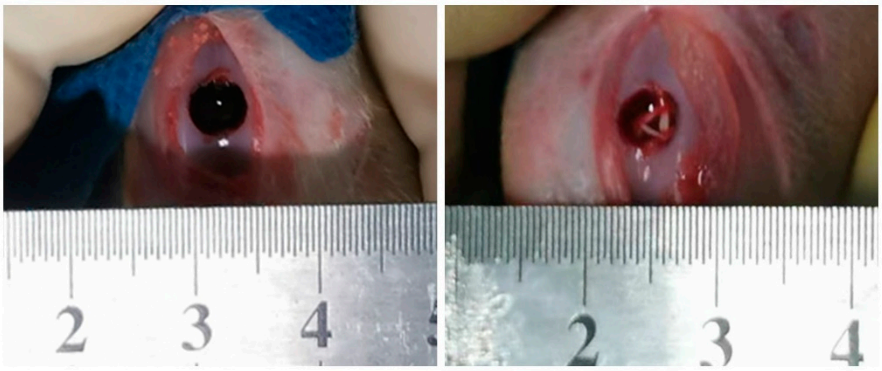

Figure S6. Typical images of (left) a rabbit knee with cartilage and subchondral bone defect and (right) scaffold implantation during the surgery.

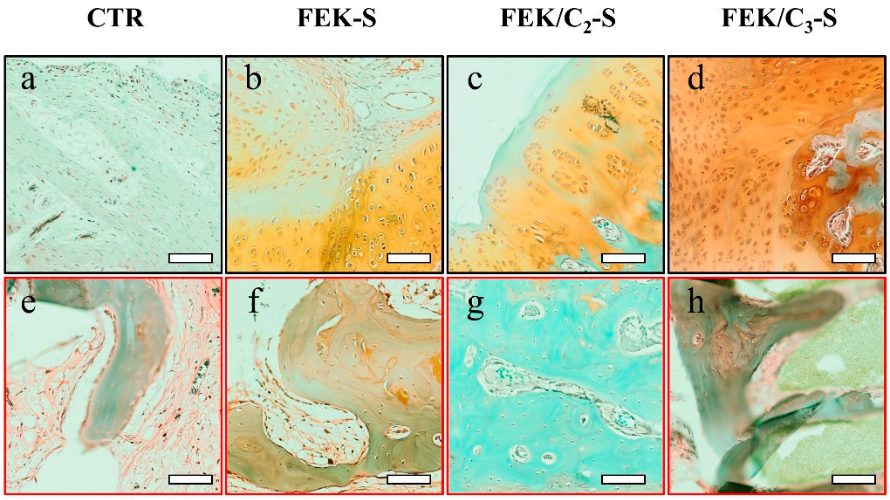

Figure S7. High magnification images of (a-d) cartilage and (e-h) subchondral bone with safranin-O/fast green staining. Scale bar: 100 μm.

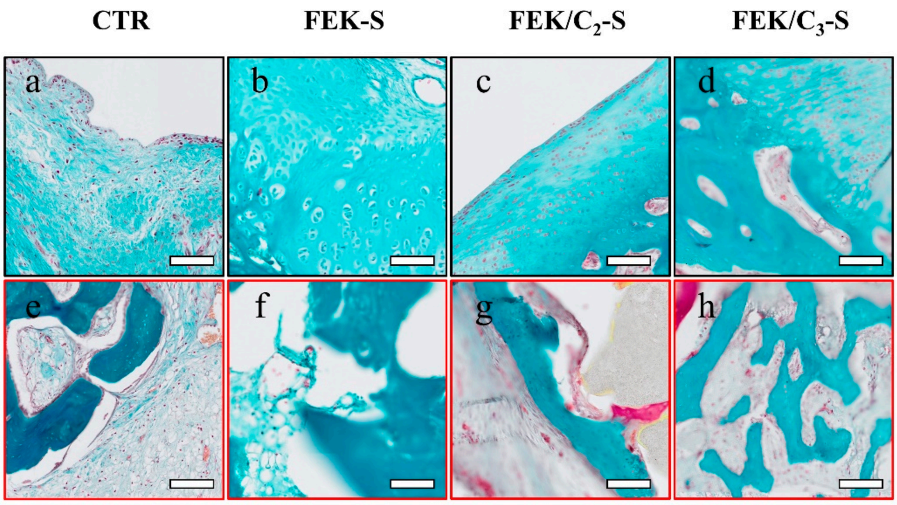

Figure S8. High magnification images of (a-d) cartilage and (e-h) subchondral bone with Masson staining. Scale bar: 100 μm.
